# Perception of social support and psychological well-being among mothers of individuals with intellectual disabilities: the mediating role of self-efficacy

**DOI:** 10.3389/fpsyt.2026.1777647

**Published:** 2026-05-13

**Authors:** Fatma Özoğlu, Emine Öztürk Karataş, Mehdi Duyan, Talip Çelik, Varol Tutal, Mehmet Ilkım, Özgür Karataş, Çetin Tan, Selçuk Okuyucu, Yunus Sadır

**Affiliations:** 1 Department of Physical Education and Sports Education for People with Disabilities, Faculty of Sports Sciences, Inönü University, Malatya, Türkiye; 2 Faculty of Health Sciences, Malatya Turgut Özal University, Malatya, Türkiye; 3 Malatya Vocational School, İnönü University, Malatya, Türkiye; 4 Faculty of Sports Sciences, Fırat University, Elâzığ, Türkiye; 5 Provincial Directorate of National Education, Erzurum, Türkiye

**Keywords:** intellectual disability, mother, perceived social support, psychological well-being, self-efficacy

## Abstract

**Background:**

The aim of this study is to examine the mediating role of self-efficacy in the relationship between social support perception and psychological well-being among mothers of individuals with intellectual disabilities.

**Methods:**

A correlational survey model was used in this study in line with the research objectives. The study group consisted of mothers of individuals with intellectual disabilities (n=80) in two central districts of Malatya province. Convenience sampling was employed in the study. The Psychological Well-being Scale was used to measure the psychological well-being of the participating mothers, the “Perceived Support Scale for Families of Children with Disabilities” was used to measure their perceived levels of social support, and the “Parental Self-Efficacy Scale” was used to measure their self-efficacy. In addition, a personal information form was used to introduce the socio-demographic characteristics of the participants. Descriptive statistics were calculated for the variables, and the normality assumption was examined using the Kolmogorov-Smirnov and Shapiro-Wilk tests. The relationships between the variables were evaluated using Pearson’s product-moment correlation analysis. Mediation analyses were performed using the PROCESS Macro (Model 4) developed by Hayes, and the significance of indirect effects was tested using the bootstrap method (5000 samples). The internal consistency reliability of the scales used in this study sample was assessed using Cronbach’s alpha coefficient.

**Results:**

There are positive and significant relationships between each of the perceived social support sub-dimensions of appreciation, informational, emotional, and companionship and self-efficacy (r = 0.28–0.36; p <0.05). A positive and significant relationship was also found between the total perceived social support score and self-efficacy (r = 0.37, p <0.01). In contrast, no direct significant relationships were found between perceived social support and its sub-dimensions and psychological well-being (p> 0.05). Psychological well-being showed only a moderate, positive and significant relationship with self-efficacy (r = 0.45, p <0.01).

**Conclusions:**

In conclusion, it can be said that the relationship between perceived social support and psychological well-being is not direct, but indirect, mediated by self-efficacy.

## 1 Introduction

Disability is not limited solely to an individual’s health condition; it is a multidimensional condition arising from the interaction of biological, psychological, social and environmental factors, encompassing physical, sensory (e.g. vision or hearing loss), intellectual, developmental, psychosocial and multiple impairments. Increasing life expectancy and the prevalence of chronic diseases are contributing to a rise in the global burden of disability (1, 2). According to data from the World Health Organisation (WHO) for 2021, over one billion people worldwide live with at least one form of disability. Approximately 1–3 per cent of these individuals fall within the category of intellectual disability (2, 3).

Intellectual disability is defined on the basis of limitations in an individual’s cognitive functions—such as reasoning, learning and problem-solving—and behavioural limitations observed in social life (4). In the American Association on Intellectual and Developmental Disabilities’ report (2018) on the definition, classification and support systems for intellectual disability, intellectual disability is described as a complex and multifaceted health condition characterised not only by cognitive limitations but also by deficiencies in the individual’s social adaptation skills and activities of daily living (5).

Every child is awaited by their parents with excitement and anxiety, and during this process, various expectations regarding the child to be born arise within the family. If the child joining the family does not show normal development, this can cause the family’s expectations to change and lead to intense anxiety and stress. Families with a child with disabilities also experience various difficulties in matters such as the child’s care, education, treatment, and upbringing.

Despite the difficulties experienced in the process of caring for children with intellectual disabilities, perceived social support emerges as a significant factor influencing the burden and levels of burnout among carers. It is reported that adequate social support reduces the burden perceived by parents (6, 7). In situations where social support is deemed insufficient, an increase in parents’ levels of depression and hopelessness is observed (7, 8). This situation poses serious risks to public mental health and has adverse effects on parents’ mental health. Although there is existing literature suggesting a theoretical link between perceived social support, care burden and burnout among mothers of children with intellectual disabilities, no study examining the relationship between these variables has been found in the literature.

The problems faced by individuals with special needs in terms of education, health, accessibility and exclusion also affect their careers in many ways. Because of these problems, having a child with special needs disrupts the psychological balance in the family system, increases the stress load on parents and, as a result, brings with it a number of social and psychological adjustment problems for parents. It can be said that parents of children with special needs have more responsibilities than parents of children without special needs. The difficulties experienced by individuals with special needs during their development, their health problems, their dependence on their parents, their parents’ concerns about their children’s future, and the lack of necessary funding can increase parental responsibilities and negatively affect them (9, 10). While children with normal development become more independent as they grow older, some children with special needs become more dependent on care and support as they grow older. At the same time, parents age and have less energy to devote to their children, which may require them to make changes in their work and life situations, causing them to feel bad (11). Parents may be unable to participate in social activities because they cannot find time for themselves due to the time spent meeting the needs of their child with special needs, and this may weaken their social ties with the community. Reduced interaction with the social environment can have a negative impact on parents (12). Social problems, such as discriminatory, exclusionary, fearful, and critical attitudes from the environment, greatly disturb parents, often causing them to avoid social relationships and become isolated (13). Exclusion, isolation, and labelling deeply affect these families (14). In contrast, healthy social support positively affects the level of acceptance of children with special needs by their families (15, 16). From an economic perspective, having special needs means requiring services in many areas, such as medical care, rehabilitation, psychological services, and education. Families are forced to meet these needs themselves due to the inadequacy of the opportunities offered by the state (17). Being unable to afford these costly services is another source of stress for families (18). In terms of lack of knowledge, many parents, regardless of their educational level, lack sufficient information about their children’s diagnosis, education, causes of disability, etc. At the same time, the social environment’s lack of knowledge about special needs also causes problems (18). From the perspective of psychological problems, parents of children with special needs often experience feelings and reactions such as shock, denial, sadness, anger, resentment, shame, depression, guilt, anxiety, unexpected crises, avoidance of the attitudes of the social environment, disappointment, and decreased self-confidence and self-respect (19). Managing these emotions and maintaining psychological well-being can sometimes be difficult and may lead to psychological disorders (20, 21). All of the above-listed problems affect mothers more within the family (22). Research frequently highlights situations where the mother takes on a large part of the responsibility for the child’s care, education and future, while the father is content to provide financial support and avoids confronting the problem (23). This situation can also be interpreted as the unequal burden of childcare being placed on mothers due to the influence of gender roles (24).

The realisation of disabilities in children with neurodevelopmental disorders such as autism, Down syndrome, cerebral palsy, and intellectual disability, either at a very early age or from birth, can negatively affect the emotional bond that may develop between mother and child. This emotional bond, which is expected to develop reciprocally as the child grows, may follow a different course for mothers of children with special needs. Faced with the increasing behavioural problems and developmental delays of the individual with special needs as they grow older, the mother may become psychologically exhausted, and as the mother becomes exhausted, more behavioural problems may emerge in the child (16, 25). Studies have shown that parents of children with special needs have lower levels of psychological well-being compared to parents of typically developing children (26). Comparative studies involving both mothers and fathers with and without children with special needs show that mothers are at greater risk in terms of psychological well-being (27). As a result, families struggle to cope with these problems and may experience psychological issues such as anxiety, anger, and even depression (28). The burden felt by the parent of a child with a disability depends on: gender, age, type and degree of disability, parents’ age, socioeconomic status, whether they live together or apart, parents’ self-efficacy, and perceived social support (29, 30). For parents, having a child with mental disabilities is one of the most challenging life experiences. In order for parents to adapt to and accept this situation, their perceptions of self-efficacy, which are part of their personal characteristics, must be high (30, 31). According to Bandura, as reported by Aksoy and Diken, perceptions of self-efficacy can influence people’s thoughts and feelings about themselves, their level of motivation towards a particular situation, and their behaviour. If individuals have high self-efficacy beliefs, they will exert more effort to accomplish a task, will not easily retreat in the face of adversity, and will be persistent and patient (31). Parental self-efficacy is defined as “a parent’s judgements and beliefs about their own capacity to perform certain tasks related to the care and upbringing of their child” (31). It is stated that mothers with high parental self-efficacy create more appropriate environmental settings for their children, resort to punishment less often, are more sensitive to their children’s reactions, and take more responsibility in their interactions with their children (30, 32). In this context, the aim of this study is to examine the relationship between perceived social support and psychological well-being variables in mothers of children with autism, Down syndrome, and mild intellectual disability, and to determine the mediating role of self-efficacy in the relationship between perceived social support and psychological well-being.

## 2 Methods

### 2.1 Design

In the study analysed using structural equation modelling, a correlational survey model was used, which aims to determine the relationship between at least two variables or more. In this model, the existence of the relationship between the variables and the nature of this relationship were examined by focusing on how the variables changed together (32). This study was designed as a cross-sectional study aimed at identifying the relationships between variables.

### 2.2 Population and sample

The population of this study consists of mothers residing in the Battalgazi and Yeşilyurt districts of Malatya province who have children with intellectual disabilities. In this study, the concept of intellectual disability was addressed based on the child being diagnosed with a disability according to the Guidance and Research Centre (RAM) assessment report and/or the health board report. In this context, mothers of children whose diagnosis was determined by official documents were included in the research population. The sample of the study consisted of a total of 80 mothers selected from the aforementioned population on a voluntary basis. Participants were reached through special education application schools, rehabilitation centres, and parent groups affiliated with these institutions operating in the districts of Battalgazi and Yeşilyurt. Considering the limited accessibility of the target group and the field conditions of the study, a convenient sampling method was used to determine the sample. The adequacy of the sample size was evaluated within the framework of the statistical analysis approach used in the study. Regression-based mediation analysis (PROCESS Macro, Model 4) was applied in the study, and in the most complex regression equation, the dependent variable is explained by two predictor variables. Considering the balance between the number of predictors and the sample size in regression analyses and the statistical robustness provided by testing indirect effects using the bootstrap method in medium-sized samples, the sample size of 80 was deemed sufficient for the analyses conducted.

### 2.3 Data collection

Ethical committee approval was obtained to carry out the study. Written and verbal consent was obtained from the mothers who agreed to participate in the study for the administration of the questionnaire. In addition, an explanatory note regarding the study was included on the first page of the data collection form to inform the mothers.

The Psychological Well-Being Scale was used to measure the psychological well-being of the mothers participating in the study, the “Perceived Support Scale for Families of Children with Disabilities” was used to measure their perceived level of social support, and the “Parental Self-Efficacy Scale” was used to measure their self-efficacy. In addition, a personal information form was used to introduce the socio-demographic characteristics of the participants.

In the study, measurement tools with previously proven validity and reliability were used to measure the social support perception scale, parental self-efficacy scale, and psychological well-being variables. The internal consistency reliability of the scales used for this research sample was evaluated using Cronbach’s alpha coefficient.

The Cronbach’s alpha coefficient for the total score of the Perceived Family Social Support Scale was calculated as.95; it was determined that the sub-dimensions of appreciation (α = .85), informational (α = .87), emotional (α = .84), and companionship (α = .84) had a high level of reliability. The Cronbach’s alpha coefficient for the Parental Self-Efficacy Scale was calculated as.91, and for the Psychological Well-Being Scale as.83. These values indicate that the measurement tools used are adequate and have a high level of reliability for the research sample.

### 2.4 Statistical analysis

The SPSS 29 package programme was used to analyse the data obtained in the study. First, descriptive statistics for the variables were calculated, and the normality assumption was examined using the Kolmogorov-Smirnov and Shapiro-Wilk tests. The relationships between the variables were evaluated using Pearson’s product-moment correlation analysis. Mediation analyses were performed using the PROCESS Macro (Model 4) developed by Hayes (33, 34), and the significance of indirect effects was tested using the bootstrap method (5000 samples).

The distribution characteristics of the variables were assessed using skewness and kurtosis coefficients. It was observed that the skewness and kurtosis values for all variables fell within the ±2 range. The literature indicates that skewness and kurtosis values within the ±2 limits are considered acceptable for normal distribution (35). These findings show that the data sufficiently meet the assumptions required for the application of parametric statistical analyses. Accordingly, parametric statistical methods were used in the analyses.

## 3 Results

The findings obtained from the research were addressed in two main sections in line with the research questions. In the first section, descriptive statistics related to the variables included in the study and the relationships between the variables were examined. In the second section, findings related to mediation analyses were presented.

### 3.1 Descriptive statistics and correlation analysis results

Pearson’s product-moment correlation analysis was applied to determine the relationships between the variables in the study. In addition, descriptive statistics for the variables were calculated. The results of these analyses are presented in **Table 1**.

**Table 1:** Relationships and descriptive statistics between the social support perception scale (SSPS), psychological well-being scale (PWBS), and parental self-efficacy scale (PSES).

Variable	1	2	3	4	5	6	7	8	9	M	SS
1. Appreciation	—									3.19	0.48
2. Informational	.80**	—								3.24	0.42
3. Emotional	.81**	.78**	—							3.11	0.52
4.Companionship	.76**	.67**	.78**	—						3.00	0.63
5. SSPS	.96**	.90**	.91**	.85**	—					3.17	0.45
6. PWBS	.36**	.33**	.35**	.28*	.37**	—				6.01	0.51
7. PSES 8. Mother Age 9. Child Age	-.02 .05 -.01	-.02 .03 .00	-.04 .04 .02	-.13 .01 -.03	-.04 .04 -.01	.45** .18 .09	— .06 .12	— .62**	—	5.45 35.84 9.14	0.55 6.21 3.41

*p <.05, **p <.001.

The demographic characteristics of the participants are presented in **Table 2**. The table contains information on educational attainment, employment status, income level, mothers’ ages, children’s ages, children’s genders and the degree of intellectual disability.

**Table 2:** Descriptive statistics.

Variable	Category	n	%
Mother’s Age Group	≤30 years	20	25.0
	31–40 years	38	47.5
	≥41 years	22	27.5
Education Level	Primary School	18	22.5
	top School	16	20.0
	High School	26	32.5
	University	20	25.0
Employment Status	Employed	34	42.5
	Unemployed	46	57.5
Income Level	Low	28	35.0
	Medium	38	47.5
	High	14	17.5
Child’s Gender	Female	36	45.0
	Male	44	55.0
Child’s Age	Early Childhood (3–6)	18	22.5
	top Childhood (7–12)	42	52.5
	Adolescence (13–18)	20	25.0
Level of Intellectual Disability	Mild	30	37.5
	Moderate	32	40.0
	Severe	18	22.5

According to the results of the Pearson correlation analysis, there are strong positive and significant correlations between the subscales of the Social Support Perception Scale (SSPS)—namely, appreciation, information, emotional support and companionship (r = .67–.81, p <.01). Furthermore, very high levels of correlation were also found between the total SSPS score and the sub-dimensions (r = .85–.96, p <.01). This indicates that the scale has strong internal consistency. Low to moderate positive and significant correlations were found between psychological well-being (PWBS) and social support variables (r = .28–.37, p <.01). This finding suggests that psychological well-being increases as perceived social support increases. The relationships between parental self-efficacy (PSES) and social support variables are weak and statistically insignificant (r ranging from -.13 to -.02). In contrast, there is a moderate, positive and significant relationship between PSES and PWBS (r = .45, p <.01). This suggests that parental self-efficacy is more closely associated with psychological well-being. When demographic variables were examined, the relationships between maternal age and the study variables were weak and non-significant (r = .01–.18). Similarly, the relationships between child age and the scales are also low (r = -.03–.12). However, a high-level positive and significant relationship was found between mother’s age and child age (r = .62, p <.01). This finding is a result that supports the expected pattern and the consistency of the sample.

### 3.2 Mediating analysis results

**Figure 1:**
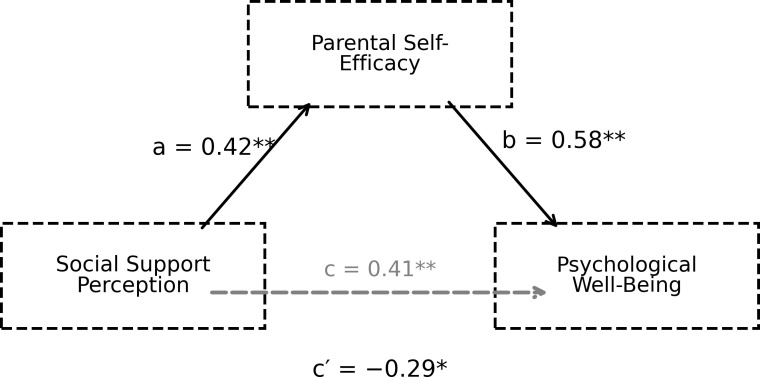
The mediating role of parental self-efficacy in the relationship between social support perception and psychological well-being.

In this study, the mediating role of parental self-efficacy in the relationship between perceived social support and psychological well-being was examined using the PROCESS Macro (Model 4). Analyses were conducted separately for the total perceived social support variable and for the appreciation, informational, emotional, and companionship sub-dimensions. In line with Hayes’ recommendations, unstandardised regression coefficients were reported in the analyses; the significance of indirect effects was assessed based on the bootstrap method (5000 resamples). The findings are reported in the tables below. The mediating role of parental self-efficacy in the relationship between perceived social support and psychological well-being is shown in **Figure 1**.

**Figure 2:**
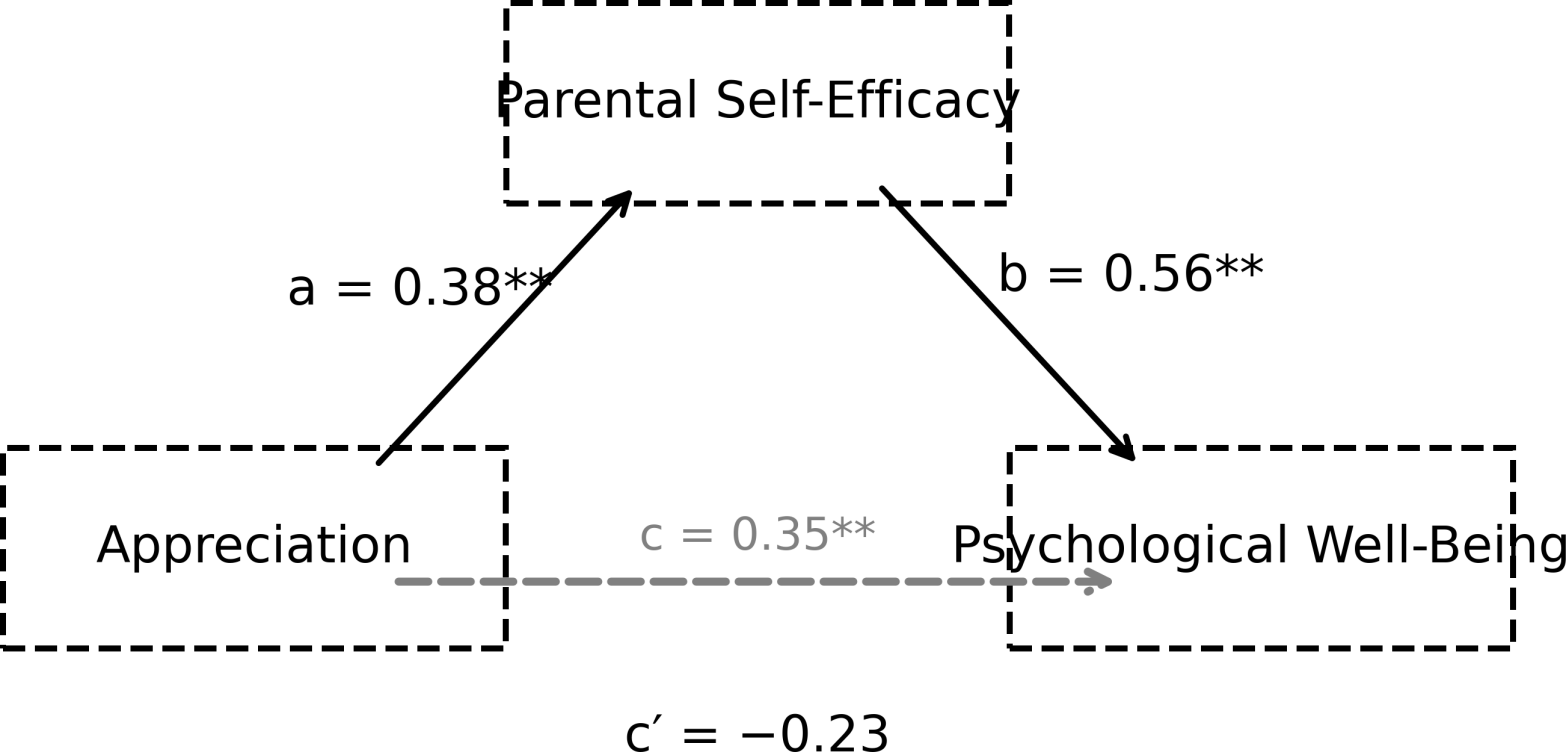
The mediating role of parental self-efficacy in the relationship between appreciation and psychological well-being.

The results of the mediation analysis in **Figure 1** indicate that the relationship between perceived social support and parental self-efficacy (path a) is positive and statistically significant (B = 0.42, SD = 0.12, p = 0.001). Similarly, the path (path b) representing the relationship between parental self-efficacy and psychological well-being is also strong and significant (B = 0.58, SD = 0.12, p < 0.001). On the other hand, the fact that the bivariate relationship between perceived social support and psychological well-being is not statistically significant (r = −0.04, *p*>.05) suggests that this relationship operates through an indirect mechanism rather than a direct linear relationship. When parental self-efficacy was included in the model, it was observed that the direction of the path (path c′) representing the direct effect of perceived social support on psychological well-being changed and became marginally significant in the negative direction (B = −0.29, SE = 0.13, p = 0.028). This finding can be attributed to the inclusion of parental self-efficacy in the model; this makes the specific variance of perceived social support on psychological well-being more apparent and creates a moderating effect between the variables. Indeed, the bootstrap analysis of the indirect effect indicates that the indirect effect of perceived social support on psychological well-being is significant (ab = 0.24, BootSH = 0.07, 95% CI [0.10, 0.38]). The results indicate that the relationship between perceived social support and psychological well-being is largely mediated by parental self-efficacy. The mediating role of the Parental Self-Efficacy Scale in the relationship between the Appreciation Subscale of Perceived Social Support and Psychological Well-being is presented in **Figure 2**.

**Figure 3:**
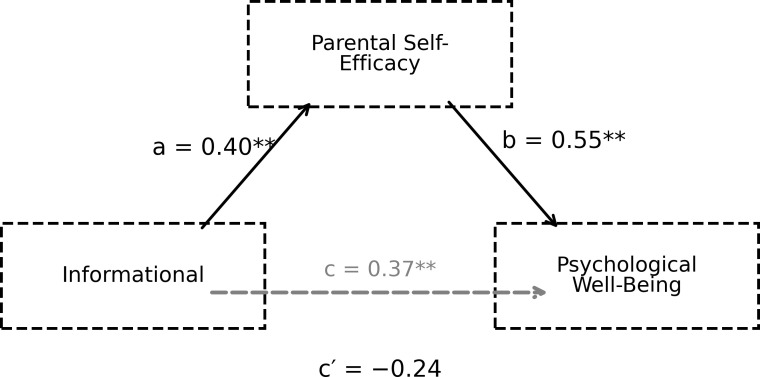
The mediating role of parental self-efficacy in the relationship between informational support and psychological well-being.

The findings presented in **Figure 2** indicate that the appreciation sub-dimension significantly predicts parental self-efficacy (path a; B = 0.38, p = 0.001) and that parental self-efficacy has a strong relationship with psychological well-being (path b; B = 0.56, p < 0.001). The direct effect of the appreciation sub-dimension on psychological well-being loses its statistical significance once the mediating variable is included in the model (path c′; p = 0.062). The bootstrap confidence interval for the indirect effect does not include zero (ab = 0.21, 95% CI [0.10, 0.34]), indicating that this relationship is largely mediated by parental self-efficacy. The mediating role of parental self-efficacy in the relationship between the informational sub-dimension of perceived social support and psychological well-being is illustrated in **Figure 3**.

**Figure 4:**
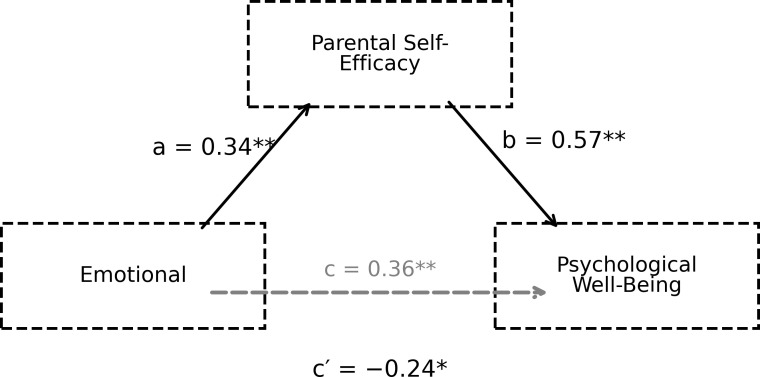
The mediating role of parental self-efficacy in the relationship between emotional support and psychological well-being.

The results presented in **Figure 3** indicate that the perception of informational social support positively and significantly predicts parental self-efficacy (path a; B = 0.40, p = 0.003). A relationship between parental self-efficacy and psychological well-being can be observed (path b; B = 0.55, p <0.001). The direct effect of the informational support sub-dimension on psychological well-being was not statistically significant (path c′; p = 0.086). However, the bootstrap confidence interval for the indirect effect excluding zero (ab = 0.22, 95% CI [0.07, 0.40]) indicates that the relationship is primarily mediated by parental self-efficacy. The mediating role of parental self-efficacy in the relationship between the emotional subscale of perceived social support and psychological well-being is presented in **Figure 4**.

**Figure 5:**
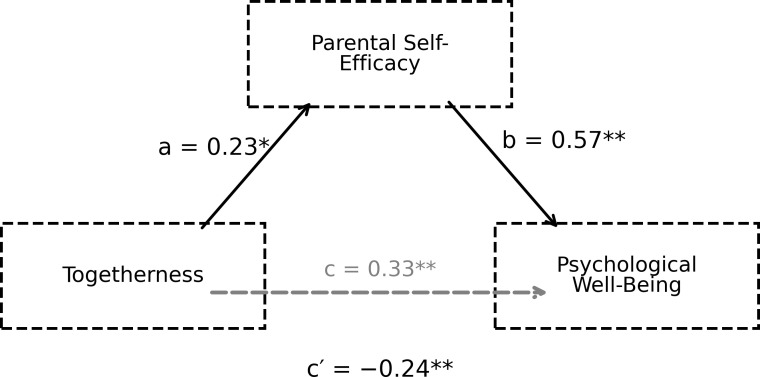
The mediating role of parental self-efficacy in the relationship between togetherness and psychological well-being.

The findings presented in **Figure 4** show that the perception of emotional support significantly predicts parental self-efficacy (path a; B = 0.34, p = 0.002) and that parental self-efficacy is a strong predictor of psychological well-being (path b; B = 0.57, p < 0.001). The relationship between perceived emotional support and psychological well-being remained significant even after a mediating variable was included in the model (path c′; B = −0.24, p = 0.041). Bootstrap results for the indirect effect (ab = 0.19, 95% CI [0.07, 0.33]) indicate that both direct and indirect pathways are involved in this relationship. The mediating role of parental self-efficacy in the relationship between the friendship sub-dimension of perceived social support and psychological well-being is presented in **Figure 5**.

According to the results presented in **Figure 5**, the ‘togetherness’ sub-dimension positively and significantly predicts parental self-efficacy (path a; B = 0.23, p = 0.012). The relationship between parental self-efficacy and psychological well-being is significant (path b; B = 0.57, p < 0.001). The direct effect of the ‘togetherness’ sub-dimension on psychological well-being remained significant when included in the model as a mediating variable (path c′; B = −0.24, p = 0.009), and the bootstrap confidence interval for the indirect effect did not include zero (ab = 0.13, 95% CI [0.02, 0.24]). These findings indicate that the relationship occurs both directly and indirectly via parental self-efficacy.

## 4 Discussion

Having a child with special needs is not easy for families. Families experience certain emotional reactions during this process and want to be understood. During this process, families need emotional, financial and informational support. This is because the families of children with special needs must be actively involved in the special education process (35). Studies conducted at national and international levels reveal that mothers of children with special needs experience high levels of stress, anger, fatigue, strain and loneliness (36). According to Kaner (37), family education is a type of effective intervention that empowers parents. As a result of this intervention, parents’ competence increases, contributing to family members’ understanding of the situation of the child with special needs and to their positive attitude towards the child, supporting them in acquiring positive coping and effective functioning skills. Social support acts as a protective factor in reducing mental health problems, and acts as a mediator in terms of protecting the individual’s mental health by enabling them to solve their problems and adapt to their environment in a balanced and healthy way (38). In their study, Fereidouni et al. (39) concluded that the quality of life of mothers with special needs children is lower than that of mothers with normally developing Children. Yüksel and Tanrıverdi (40) emphasised in their study that families need to be supported with multi-faceted social programmes to cope with social isolation and exclusion. There are numerous studies in the literature on parents of children with special needs. These studies reveal that parents face multidimensional stress factors such as inadequate health, education, and social support, social prejudices, and economic difficulties during the care process.

Factors such as the care, education, and health services required in the life of a child with special needs have been seen to have significant effects on the mental health of the mother and father. Recent studies have shown that social support not only directly improves psychological well-being but also strengthens a person’s internal resources. For example, the protective role of social support in the relationship between the burden of caring for the family and psychological well-being has been emphasised. Furthermore, it has been reported that a high perception of social support improves well-being by reducing caregivers’ stress and depressive moods (41).

Parents’ constant anxiety about the future can significantly affect their stress levels, and the emotional strain experienced can lead to various problems in relationships within the home (42). In this context, our research findings are consistent with the existing scientific literature that recognises the importance of social support networks, self-efficacy, and psychological well-being. The study conducted by Vural Yüzbaşı (44) noted that, although demographic variables such as age and gender may lead to differences in the subdimensions of well-being, they do not have a direct determinative effect on well-being. A study conducted by Kaymakcı et al. (2022) found that the mothers’ age, gender, number of children, and the time and duration of the child’s diagnosis had no significant effect on the social support perceived by the mothers (45). Studies examining the relationship between parents’ ages and their perceptions of social support and well-being present a variety of findings in the literature. The fact that findings in the literature regarding the relationship between parents’ ages and their levels of life satisfaction vary considerably suggests that the age variable should be assessed in conjunction with external factors—such as educational background, income levels and access to social support—as well as internal factors—such as stress, depression and marital status—in terms of life satisfaction. Rather than being merely a predictor variable, age can be considered a factor whose effect emerges in conjunction with other variables within a model. Coleman and Karraker (2000) report that there is no relationship between parental self-efficacy and parental age (46). Similarly, Al-Kandari and Al-Quashan (2010) stated that there is no relationship between age and parental self-efficacy. However, when considering the theoretical framework of parental self-efficacy, it is expected that young mothers would have lower levels of parental self-efficacy (47). Al-Kandari and Al-Qashan (2010) also report that parents’ self-efficacy does not differ according to gender or age, but does differ according to diagnosis. Rather than being a predictor variable in its own right, age can be considered within the model as a factor whose effect emerges in conjunction with other variables (47).

Sharifian and colleagues’ (43) study with mothers of children with disabilities found that psychological resilience training reduced mothers’ stress levels and increased their resilience. Rambod and colleagues’ (48) study found that hope plays a mediating role in the relationship between resilience and depression, anxiety, and stress. Hafızoğlu and Koç’s (49) research showed that the parent’s level of psychological resilience is related to the children’s attention and anxiety levels and may indirectly affect parental stress.

According to Coşkun and Akkaş (50), as the social support levels of mothers with disabled children increase, their anxiety levels decrease. Studies have shown that when families’ needs are not met through expert support and assistance, their stress and anxiety levels increase, but when their needs are met, their stress is alleviated, they feel more comfortable, and they can contribute more to their children’s education (51). According to the theoretical framework developed by Albanese et al. (52), parental self-efficacy is considered an important internal resource that reduces negative psychological outcomes such as stress, depression, and fatigue and increases well-being.

In her study, Goretty (53) examined the mediating role of parental self-efficacy in the relationship between social support and subjective well-being among mothers providing home education affiliated with PKBM Y. She found that social support significantly predicted parental self-efficacy, which in turn predicted life satisfaction and positive and negative emotions. Social support also had direct effects on all three well-being components. Indirect effects were significant for life satisfaction, positive emotions, and negative emotions, indicating partial mediation (53). This is consistent with the findings of our study. The current literature indicates that mothers of children with disabilities are at psychological risk due to caregiving responsibilities, uncertainty, social stigma, and economic burden, and therefore require greater social support. Social support helps mothers realise that the challenges they face are surmountable by enabling them to share their feelings and receive information and practical assistance. However, recent research indicates that the effect of social support on psychological well-being is generally indirect rather than direct. This study confirms this view by showing that parents’ self-efficacy mediates the effect of social support on psychological well-being, a finding that is gaining increasing recognition in the literature.

### 4.1 Strengths and limitations

The most significant strength of this research is its examination of parents’ self-efficacy as a mediator between parents’ perceived social support and their mothers’ mental health. It examines the self-efficacy of parents of children with intellectual disabilities and offers a process-oriented explanation of caregivers’ well-being. Its focus on a high-risk and underrepresented group strengthens the clinical relevance of the findings. However, the cross-sectional nature of the study limits inferences about causality, and relying solely on self-report measures may introduce variability in commonly used measures. Furthermore, the exclusion of clinical variables related to disability (such as time to diagnosis, comorbidities, clinical severity, and treatment history) constitutes a significant limitation. The sample comprising only mothers further limits the generalizability of the findings to other caregivers and disability groups. Longitudinal and multi-method studies are needed to validate and extend these findings.

### 4.2 Conclusion

The results of this study indicate that the relationship between perceived social support and mental health is largely determined by parents’ own self-efficacy. In recent studies with mothers of children with disabilities in the literature, evidence of the mediating role of parental self-efficacy is increasing. Recent studies have revealed that variables such as parental self-efficacy, psychological resilience, and coping skills play a mediating role in the relationship between social support and psychological well-being. By focusing on parental self-efficacy, this research makes an important contribution to the literature and provides a comprehensive framework for explaining psychological well-being.

In this context, we can conclude that measures aimed at improving the mental health of mothers with intellectual disabilities should include strategies that encourage the development of personal skills and thus increase their self-efficacy. These measures should not focus solely on strengthening social support networks. Social support programmes yield more effective results when combined with education, skill development modules that strengthen confidence in self-efficacy, or psychological techniques that encourage personal empowerment.

Psychological and social support programmes for mothers of individuals with intellectual disabilities should not only provide social support but also include skill-based content designed to strengthen parents’ confidence in their parenting abilities.

Mental health programmes focusing on problem solving, stress management and the development of parenting skills should be easily accessible.

When the mother of a child with intellectual disabilities is identified as being at psychological risk, she should be offered comprehensive psychological counselling and preventive support services.

The mental health of parents of children with intellectual disabilities should be regularly monitored as part of the services provided by guidance and awareness centres, special education institutions, and local authorities. Comprehensive service models should be developed to increase parents’ confidence.

## Data Availability

The original contributions presented in the study are included in the article/supplementary material. Further inquiries can be directed to the corresponding author.

## References

[1] Pađen L Vettorazzi R Ravljen M Pajnič M . Slovenian nursing students' perspectives and experiences with nursing care of people with intellectual and developmental disabilities: a qualitative descriptive study. Healthcare. (2025) 13:61. doi: 10.3390/healthcare13010061. PMID: 39791668 PMC11720056

[2] World Health Organization (WHO) . Disability. Geneva: WHO (2021). Available online at: https://www.who.int/health-topics/disability#tab=tab_1 (Accessed December 20, 2025).

[3] McBride O Heslop P Glover G Taggart T Hanna-Trainor L Shevlin M . Prevalence estimation of intellectual disability using national administrative and household survey data: the importance of survey question specificity. Int J Popul Data Sci. (2021) 6:1342. doi: 10.23889/ijpds.v6i1.1342. PMID: 34164584 PMC8188522

[4] American Psychiatric Association . Diagnostic and statistical manual of mental disorders. Arlington, VA: American Psychiatric Association (2013).

[5] American Association on Intellectual and Developmental Disabilities . Intellectual disability: definition, diagnosis, classification, and systems of supports. Silver Spring, MD: AAIDD (2018). Available online at: https://www.aaidd.org/docs/default-source/default-document-library/definition-diagnosis-classification-and-systems-of-supports-(12e).pdf (Accessed December 20, 2025).

[6] Gönültaş N . Zihinsel ve fiziksel engelli çocuğa sahip ebeveynlerde algılanan sosyal destek ile bakım verme yükü arasındaki ilişkinin incelenmesi. İstanbul: Beykent Üniversitesi, Sosyal Bilimler Enstitüsü (2017).

[7] Şahin Varol H Altun Yılmaz E . Engelli çocuğu olan annelerin depresif semptomlar ve algıladıkları sosyal destek açısından incelenmesi. Hemşirelik Bilimi Dergisi. (2023) 6:97–105. doi: 10.54189/hbd.1305082

[8] Karadağ G . Engelli çocuğa sahip annelerin yaşadıkları güçlükler ile aileden algıladıkları sosyal destek ve umutsuzluk düzeyleri. TAF Prev Med Bull. (2009) 8:315–22.

[9] Zulfia R . Mothers' experience in caring for children with special needs: a literature review. Indones J Disabil Stud. (2020) 7:8–18. doi: 10.21776/ub.ijds.2019.007.01.2

[10] Colak B Kahriman I . Evaluation of family burden and quality of life of parents with children with disability. Am J Fam Ther. (2023) 51:113–33. doi: 10.1080/01926187.2021.1941421. PMID: 37339054

[11] Kaya O Yondem D . Otizmli çocuğu olan annelerde psiko-eğitim grup programının algılanan stres düzeyine etkisi. Uşak Univ Eğit Araşt Derg. (2020) 6:20–33.

[12] Ulugbek Usmonovich O Temirpulatovich TB . The influence of the presence of mentally ill children in the family on the psyche of parents. J Educ Ethics Values. (2023) 2:8.

[13] Celik R . Adalet, kapsayıcılık ve eğitimde hakkaniyetli fırsat eşitliği. Ankara: Pegem Akademi (2021).

[14] Cantero-Garlito PA Moruno-Miralles P Flores-Martos JA . Mothers who take care of children with disabilities in rural areas of a Spanish region. Int J Environ Res Public Health. (2020) 17:2766. doi: 10.3390/ijerph17082920. PMID: 32340226 PMC7215576

[15] Çetin K . Engelli çocuklara sahip ailelerin çocuklarını kabul-reddi ile sosyal destek ilişkisinin çeşitli değişkenlerce yordanması. J Educ Sci. (2018) 9:1–15.

[16] Lu M Chen J He W Pang F Zou Y . Association between perceived social support of parents and emotional/behavioral problems in children with ASD: a chain mediation model. Res Dev Disabil. (2021) 113:103943. doi: 10.1016/j.ridd.2021.103933. PMID: 33730685

[17] Oral A Aydın R Ketenci A Akyüz G Sindel D Yalıman A . Dünya Engellilik Raporu: Türkiye'de engellilik ile ilgili konuların analizi ve fiziksel tıp ve rehabilitasyon tıp uzmanlığının katkıları. Turk J Phys Med Rehabil. (2016) 62:83–97. doi: 10.5606/tftrd.2016.219. PMID: 24283811

[18] Kot M Sönmez S Eratay E . Özel gereksinimli bireylere sahip ailelerin yaşadıkları zorluklar. Abant İzzet Baysal Üniv Eğitim Fak Derg. (2018) 18:1455–74.

[19] Shenaar-Golan V . The subjective well-being of parents of children with developmental disabilities: the role of hope as predictor and fosterer of well-being. J Soc Work Disabil Rehabil. (2016) 15:77–95. doi: 10.1080/1536710X.2016.1162119. PMID: 26959099

[20] Farajzadeh A Maroufizadeh S Amini M . Factors associated with quality of life among mothers of children with cerebral palsy. Int J Nurs Pract. (2020) 26:e12811. doi: 10.1111/ijn.12811. PMID: 31981299

[21] Hemati Alamdarloo G Majidi F . Feelings of hopelessness in mothers of children with neurodevelopmental disorders. Int J Dev Disabil. (2022) 68:485–94. doi: 10.1080/20473869.2020.1736886. PMID: 35937174 PMC9351562

[22] Bourke-Taylor HM Lee DCA Tirlea L . Interventions to improve mental health of mothers of children with disability. J Autism Dev Disord. (2021) 51:3690–706. doi: 10.1007/s10803-020-04826-4. PMID: 33389452

[23] Hickey EJ Hartley SL Papp L . Psychological well-being and parent–child relationship quality in relation to child autism. Fam Process. (2020) 59:636–50. doi: 10.1111/famp.12432. PMID: 30844091 PMC6732055

[24] Işık A Akbaş E . Özel gereksinimli çocuklara sahip ailelerin evlilik yaşamına toplumsal cinsiyet odaklı yaklaşım. Soc Sci Res J. (2019) 8:120–35.

[25] Öz B Yüksel T Nasiroğlu S . Depression–anxiety symptoms and stigma perception in mothers of children with autism spectrum disorder. Noropsikiyatri Ars. (2020) 57:50–5. doi: 10.29399/npa.23655. PMID: 32110151 PMC7024823

[26] Eraslan-Çapan B Tümlü C . Engelli çocuğa sahip ebeveynlerin duygusal sağırlık ve psikolojik iyi oluş düzeylerinin incelenmesi. Kalem Int J Educ Hum Sci. (2018) 15:493–518. doi: 10.23863/kalem.2019.112

[27] Hallberg U . Differences in health and well-being of parents of children with disabilities. Int J Qual Stud Health Well-being. (2014) 9:23164. doi: 10.3402/qhw.v9.24343. PMID: 24746248 PMC3991835

[28] Palancı M . A prediction of the resilience, subjective well-being and marital adjustment of the parents having children with disabilities based on psycho-social competence. Egitim ve Bilim. (2018) 43:217–36. doi: 10.15390/EB.2017.4384

[29] Yıldırım Sarı H . Zihinsel engelli çocuğu olan ailelerde aile yüklenmesi. Cumhuriyet Üniv Hemşirelik Yüksekokulu Derg. (2007) 11:1–10.

[30] Çetin F Okan H Basım YN . Psikolojik dayanıklılığın açıklanmasında beş faktör kişilik özelliklerinin rolü: Bir kanonik ilişki analizi. Türk Psikoloji Derg. (2015) 30:65–78.

[31] Aksoy A Diken İH . Annelerin ebeveynlik öz yeterlik algıları ile gelişimi risk altında olan bebeklerin gelişimleri arasındaki ilişkiyi inceleyen araştırmalara bir bakış. Ankara Univ Özel Eğit Derg. (2017) 18:455–78.

[32] Karasar N . Bilimsel araştırma yöntemi: kavramlar, ilkeler, teknikler. Ankara: Nobel Yayın Dağıtım (2023).

[33] Hayes AF . Introduction to mediation, moderation, and conditional process analysis. New York: The Guilford Press (2022).

[34] George D Mallery P . SPSS for Windows step by step. Boston: Pearson (2010).

[35] Arslan A Ulaş AH Coşkun MK . Özel eğitimde aile eğitimine yönelik bir derleme çalışması. Eğitim Kuram ve Uyg Araşt Derg. (2020) 6:356–72.

[36] Ören B Aydın R . Engelli çocuğa sahip ebeveynlerde bakım veren yükü ve depresyon. CBÜ Sağlık Bil Enst Derg. (2020) 7:302–9. doi: 10.34087/cbusbed.682392

[37] Kaner S . Aile katılımı ve işbirliği. In: Sucuoğlu B , editor.Zihin engelliler ve eğitimleri. Kök Yayıncılık, Ankara (2015). p. 355–402.

[38] Özdemir B . İlkokul öğrencilerinin algıladığı sosyal destek düzeyi ve çözüm odaklı psiko-eğitim programının etkisi. Ankara: Gazi Üniversitesi (2022).

[39] Fereidouni Z Kamyab AH Dehghan A Khiyali Z Ziapour A Mehedi N . A comparative study on the quality of life and resilience of mothers with disabled and neurotypically developing children in Iran. Heliyon. (2021) 7:e07172. doi: 10.1016/j.heliyon.2021.e07285. PMID: 34222686 PMC8243004

[40] Yüksel H Tanrıverdi A . Özel gereksinimli çocuğa sahip ailelerin yaşadıkları sosyal sorunlar ve baş etme yolları. Ankara Univ Özel Eğit Derg. (2019) 20:535–59. doi: 10.21565/ozelegitimdergisi.493089

[41] Bongelli R Busilacchi G Pacifico A Fabiani M Guarascio C Sofritti F . Caregiving burden, social support, and psychological well-being among family caregivers of older Italians: a cross-sectional study. Front Public Health. (2024) 12:1474967:1326265. doi: 10.3389/fpubh.2024.1474967. PMID: 39507659 PMC11537916

[42] Avşaroğlu S Okutan H . Zihin engelli çocuğu olan ailelerin yaşam doyumları ve psikolojik belirtileri. Manas J Soc Stud. (2018) 7:421–38.

[43] Sharifian P Kuchaki Z Shoghi M . Effect of resilience training on stress, hope and psychological toughness of mothers living with mentally and physically disabled children. BMC Pediatr. (2024) 24:142. doi: 10.1186/s12887-024-04828-6. PMID: 38778364 PMC11110178

[44] Vural Yüzbaşı D . Zihinsel engelli çocuğa sahip annelerin ıyi oluşlarinin başa çikma tarzları, aile gereksinimleri ve sosyal destek değişkenleriyle modellenmesi. Celal Bayar Üniversitesi Sos Bilim Derg. (2019) 17:107–34. doi: 10.18026/cbayarsos.467127

[45] Kaymakçı D Ekşi H Yaman GK . The mediating role of emotion regulation on the effect of parental pressure for greater academic performance on gaming addiction. Turkish psychol Couns Guidance J. (2022) 12:495–512. doi: 10.17066/tpdrd.1175196

[46] Coleman PK Karraker KH . Parenting self-efficacy among mothers of school-age children: conceptualization, measurement, and correlates. Family Relations: Interdiscip J Appl Family Stud. (2000) 49:13–24. doi: 10.1111/j.1741-3729.2000.00013.x. PMID: 40046247

[47] Al-Kandari HY Al-Qashan H . Maternal self-efficacy of mothers of children with intellectual developmental disabilities down syndrome and autism in Kuwait. Childand Adolesc Soc Work J. (2010) 27:21–39. doi: 10.1007/S10560-009-0189-6. PMID: 30311153

[48] Rambod M Nassabeh F Salmanpour M Pasyar N . The mediation role of hope in the relationship of resilience with depression, anxiety, and stress in caregivers of children and adolescents with cancer. Sci Rep. (2024) 14:1225. doi: 10.1038/s41598-024-65922-4. PMID: 38982133 PMC11233650

[49] Hafızoğlu M Koç V . Aile ilişkilerinin ve ebeveynin olumsuz çocukluk çağı yaşantıları ile psikolojik sağlamlığının çocukların dikkat ve kaygı düzeyi ile ilişkisi. İletişim ve Toplum Araşt Derg. (2025) 5:57–81. doi: 10.59534/jcss.1468905

[50] Coşkun Y Akkaş G . Engelli çocuğu olan annelerin sürekli kaygı düzeyleri ile sosyal destek algıları. Ahi Evran Univ Kırşehir Eğit Fak Derg. (2009) 10:213–27.

[51] Çetrez İşcan G Malkoç A . Özel gereksinimli çocuğa sahip ailelerin umut düzeylerinin başa çıkma yeterliği ve yılmazlık açısından incelenmesi. Ege Eğitim Derg. (2017) 18:564–93. doi: 10.12984/egeefd.305928

[52] Albanese AM Russo GR Geller PA . The role of parental self-efficacy in parent and child well-being: A systematic review of associated outcomes. Child Care Health Dev. (2019) 45:333–63. doi: 10.1111/cch.12661. PMID: 30870584

[53] Goretty M . Parenting self-efficacy as a mediator between social support and mothers' well-being. J Psikoborneo. (2025) 13:348–56. doi: 10.30872/psikoborneo.v13i2

